# LRRK2 G2019S Promotes Colon Cancer Potentially via LRRK2–GSDMD Axis-Mediated Gut Inflammation

**DOI:** 10.3390/cells13070565

**Published:** 2024-03-23

**Authors:** Yuhang Wang, Joyce Z. Gao, Taylor Sakaguchi, Thorsten Maretzky, Prajwal Gurung, Nandakumar S. Narayanan, Sarah Short, Yiqin Xiong, Zizhen Kang

**Affiliations:** 1 Department of Pathology, University of Iowa, Iowa City, IA 52242, USA; 2 Department of Internal Medicine, University of Iowa, Iowa City, IA 52242, USA; 3 Iowa Neuroscience Institute, University of Iowa, Iowa City, IA 52242, USA; 4 Department of Neurology, University of Iowa, Iowa City, IA 52242, USA

**Keywords:** LRRK2 G2019S, colitis, colon cancer, inflammation

## Abstract

Leucine-rich repeat kinase 2 (LRRK2) is a serine–threonine protein kinase belonging to the ROCO protein family. Within the kinase domain of LRRK2, a point mutation known as LRRK2 G2019S has emerged as the most prevalent variant associated with Parkinson’s disease. Recent clinical studies have indicated that G2019S carriers have an elevated risk of cancers, including colon cancer. Despite this observation, the underlying mechanisms linking LRRK2 G2019S to colon cancer remain elusive. In this study, employing a colitis-associated cancer (CAC) model and LRRK2 G2019S knock-in (KI) mouse model, we demonstrate that LRRK2 G2019S promotes the pathogenesis of colon cancer, characterized by increased tumor number and size in KI mice. Furthermore, LRRK2 G2019S enhances intestinal epithelial cell proliferation and inflammation within the tumor microenvironment. Mechanistically, KI mice exhibit heightened susceptibility to DSS-induced colitis, with inhibition of LRRK2 kinase activity ameliorating colitis severity and CAC progression. Our investigation also reveals that LRRK2 G2019S promotes inflammasome activation and exacerbates gut epithelium necrosis in the colitis model. Notably, GSDMD inhibitors attenuate colitis in LRRK2 G2019S KI mice. Taken together, our findings offer experimental evidence indicating that the gain-of-kinase activity in LRRK2 promotes colorectal tumorigenesis, suggesting LRRK2 as a potential therapeutic target in colon cancer patients exhibiting hyper LRRK2 kinase activity.

## 1. Introduction

Leucine-rich repeat kinase 2 (LRRK2) stands as a pivotal serine–threonine protein kinase within the expansive ROCO protein family, characterized by its multifaceted structure encompassing distinct domains. Among these domains are the N-terminal armadillo domain, ankyrin repeats, leucine-rich repeat (LRR) domain, Ras of complex protein domain (Roc) GTPase, C-terminal of Roc (COR) domain, kinase domain and a C-terminal WD40 domain [1,2]. The breadth of LRRK2 functions extends across diverse cellular processes, including autophagy, lysosome function, endocytosis and modulation of the Golgi network [3,4,5]. Recent insights have underscored LRRK2’s crucial involvement in immune regulation [6,7]. We previously identified LRRK2 as a novel modulator of the activation of the NLRC4 inflammasome [8]. Of note, LRRK2 was first identified in 2004 as a genetic cause of Parkinson’s disease (PD) [9,10]. Subsequently, LRRK2 mutations were also discovered to be linked with inflammatory bowel disease (IBD) [11,12,13], cancer, leprosy, etc. [14,15]. Among these, gain-of-kinase-activity mutant LRRK2 G2019S is the most prevalent variant associated with PD [16,17]. Intriguingly, several previous studies have demonstrated that LRRK2 G2019S mutation carriers have an overall elevated risk of cancer, especially hormone-related cancers, and breast cancer in women [18,19,20]. Recent studies have further suggested that LRRK2 G2019S PD patients exhibit drastically increased risks of colon cancer and leukemia compared with idiopathic PD patients [21,22]. Despite these revelations, the underlying mechanisms of LRRK2 G2019S-mediated tumorigenesis remain largely elusive, necessitating empirical scrutiny into its role in colon cancer development.

Colorectal cancer is the third most prevalent cancer worldwide [21]. Inflammation is recognized as a hallmark of cancer development and progression [23]. In humans, the correlation between IBD and colorectal cancer has long been established. Colitis-associated colorectal cancer (CAC) may affect as many as 18.4% at 30 years after the onset of ulcerative colitis [24], which is 2- to 3-fold that of the general population [25]. Furthermore, CAC patients have a worse prognosis than colorectal cancer patients without a history of IBD [26,27]. Increased mortality rates for CAC have also been reported [28,29]. While molecular mechanisms underlying colorectal cancer remain unclear, inflammation is considered a driving force for the pathogenesis [30]. Azoxymethane/dextran sulfate sodium (AOM/DSS)-induced colon cancer in mice is a widely used animal model for investigating the pathophysiology of CAC [31,32,33].

Expressed across various cellular subsets, including myeloid cells, B cells, T cells, microglial cells and epithelial cells [34,35], LRRK2 upregulation in the lamina propria of intestinal biopsies from Crohn’s disease(CD) patients underscores its pivotal role in intestinal inflammation [36]. Genome-wide association studies (GWASs) have further underscored the association between the LRRK2 locus and IBD [37,38], with exome sequencing revealing shared LRRK2 alleles in CD and PD, including the gain-of-function variant N2081D within the LRRK2 kinase domain, suggesting excessive kinase activity might be crucial for the pathogenesis [39,40,41]. Therefore, we hypothesized that LRRK2 G2019S promotes the development of colorectal cancer by fostering intestinal inflammation. The objective of this study was to elucidate how gain-of-kinase activity mutant LRRK2 G2019S promotes colon tumorigenesis in a colitis-associated cancer model.

In the present article, we delineate the role of LRRK2 G2019S in promoting colon tumorigenesis within a mouse model of CAC. Moreover, we elucidate how LRRK2 G2019S heightens the susceptibility to DSS-induced colitis in mice. Mechanistically, we find that LRRK2 G201S significantly amplifies inflammasome activation, instigates epithelial cell necrosis and stimulates the production of reactive oxygen species within the gut. Notably, the kinase activity of LRRK2 emerges as pivotal for these regulatory functions. Thus, our experimental findings unequivocally underscore the pro-tumorigenic implications of LRRK2 G2019S in the colorectal cancer pathogenesis.

## 2. Materials and Methods

### 2.1. Animals

LRRK2 G2019S KI (LRRK2 KI) mice [42] were all purchased from Jackson Laboratory (JAX030961). The LRRK2 KI strain is congenic on the C57BL/6J background. To breed the littermate wild-type control mice, LRRK2^KI/+^ male mice were mated with LRRK2^KI/+^ female mice, and sex-matched LRRK2^+/+^ mice (referred to as WT control) and LRRK2^KI/KI^ mice (referred to as LRRK2 KI) were used at the age of 8–12 weeks. Both male and female mice were used for the experiments. All mice were bred and maintained in individually ventilated cages under specific pathogen-free conditions in accredited animal facilities. The animal experiments were approved by the Institutional Animal Care and Use Committee of the University of Iowa (IACUC#: 0112357).

### 2.2. DSS-Induced Acute Colitis

Colitis was induced by treating mice with 2.5% DSS for 7 days as described previously [43]. In brief, sex-matched LRRK2 WT and LRRK2 G2019S KI mice at the age of 8–12 weeks were given 2.5% DSS (MP Biomedical) in normal drinking water for 7 days, followed with regular drinking water from the animal facility for another 2 days, to induce acute colitis. Mouse weights were monitored every day during the colitis model. The humane endpoint for the DSS study, in accordance with Institutional Animal Care and Use Committee recommendations from the University of Iowa, was the loss of 20% of the initial body weight.

### 2.3. Disease Activity Index (DAI)

The disease activity index (DAI) was calculated with minor adjustments to the methods previously described [44,45]. In brief, the DAI score, a composite measure, is derived from evaluations of weight loss, stool consistency and rectal bleeding, with scores ranging from 0 (indicating no disease) to 10 (indicative of severe colitis). The scoring criteria are as follows: (a) Weight loss is scored from 0 (no loss) to 4, with 1 point for a 1–5% loss, 2 points for a 5–10% loss, 3 points for a 10–15% loss, and 4 points for more than a 15% loss. (b) Stool consistency scores range from 0 (normal) to 3, with 1 point for soft stools, 2 points for very soft stools, and 3 points for watery diarrhea. (c) Bleeding is scored from 0 (no bleeding) to 3, with 1 point for slight bleeding (hemoccult positive), 2 points for hemoccult positivity and visible pellet bleeding, and 3 points for gross bleeding. Fecal occult blood was detected using the Hemoccult Single Slides Rapid Diagnostic Test Kit (Catalog No. 60151A, Beckman Coulter, Brea, CA, USA).

### 2.4. AOM/DSS-Induced Colon Cancer

Colonic tumors were induced using the azoxymethane/dextran sulfate sodium (AOM/DSS) protocol as described previously [31,46]. Male and female mice aged 8–12 weeks were utilized for this study. The mice were initially injected intraperitoneally with AOM at a dose of 10 mg/kg body weight. On day 3 after AOM administration, the mice were exposed to 2.5% DSS in their drinking water for 5 consecutive days. This was followed by a resting period of 16 days. This cycle was repeated another two times. Tumorigenesis assessment was performed on day 65 following the initial AOM injection. For tumor analysis, after mouse euthanasia, the colon was longitudinally cut and then fixed in 10% neutral buffered formalin overnight. All colon tumors were carefully counted and measured using a stereo microscope. Representative tumors were selected for paraffin embedding and sectioning at a thickness of 5 μm. Histological analyses were conducted by hematoxylin and eosin (H&E) staining.

### 2.5. LRRK2 and GSDMD Inhibitor Administration

The LRRK2 kinase inhibitor LRRK2-IN-1 (MedChemExpress, Monmouth Junction, NJ, USA) was reconstituted in corn oil as described previously [47]. Experimental groups of mice were treated with LRRK2-IN-1 by intraperitoneal (i.p.) injection once a day at a dose of 100 mg/kg body weight. GSDMD inhibitors diroximel fumarate (DMF, Sigma-Aldrich, Darmstadt, Germany) and disulfiram (Sigma-Aldrich, Darmstadt, Germany) were administered by oral gavage and i.p. injection once a day, respectively, both at doses of 50 mg/kg [48,49]. The inhibitor treatments were administered concurrently with the DSS treatment as specified in the experiments.

### 2.6. Immunoblot

Tissues were homogenized using a radioimmunoprecipitation (RIPA) assay buffer, which was supplemented with the Complete Mini Protease Inhibitor Cocktail and Phosphatase Inhibitor Cocktail from Roche. The lysates were put on ice for a period of 30 min and vortexed every 5 min. Following centrifugation at 15,000 rpm for 15 min at 4 °C, the supernatants were collected. The protein concentration was determined by a BCA Protein Assay Kit from Pierce. Subsequently, the proteins were resolved by SDS-PAGE and transferred to a 0.45 mm PVDF membrane. For immunoblot analysis, the indicated primary antibodies are listed in Supplemental Table S1 and were used at a 1000-fold dilution. Horseradish peroxidase (HRP)-conjugated secondary antibodies were used depending on the host species of the primary antibodies.

### 2.7. ELISA

Whole colon cultures derived from DSS-treated mice were used to assess cytokine production using ELISA kits obtained from R&D Systems (Minneapolis, MN, USA), following the manufacturer’s instructions. The following catalog numbers were used for specific cytokines: TNF (DY410-05), IL-1β (DY401-050), IL-6 (DY406-05) and IL-18 (7625-05). Cytokine levels were normalized to the weight of the colon tissue used.

### 2.8. Intestinal Permeability Analysis

Intestinal permeability in vivo was assessed using an FITC-dextran (average molecular mass 4000 kDa; Sigma-Aldrich) gavage experiment, both in naive conditions and during colitis. Briefly, mice were fasted overnight prior to gavage of FITC-dextran at a dosage of 60 mg per 100 g of body weight. After 4 h, blood samples were collected via cardiac puncture and sera were obtained by centrifugation at 10,000× *g* for 10 min. The fluorescence intensity of the serum samples was measured using a SpectraMax i3 instrument from Molecular Devices (San Jose, CA, USA). The concentration of FITC-dextran was determined by referencing a standard curve generated from serial dilutions of FITC-dextran.

### 2.9. Isolation of Intestinal Epithelial Cells (IECs)

IECs were isolated as described previously [50]. The procedure involved gently extracting the large intestine from the abdominal cavity, followed by the removal of mesentery and fatty tissue using forceps. The large intestine, excluding the cecum, was dissected and longitudinally opened, then cut into 1 cm pieces. The intestinal pieces were thoroughly washed three times with ice-cold PBS. Subsequently, the pieces were further chopped into 5 mm fragments and incubated in 5 mM EDTA/PBS solution at 4 °C on a rocking platform for 30 min. IECs were released by shaking the tubes for 2 min, and then collected by centrifugation at 200× *g* for 10 min at 4 °C. The dissociated cells were washed and resuspended in PBS with 10% FBS.

### 2.10. Total ROS and Mitochondrial ROS Measurement

To assess the total ROS in IECs, the cells were incubated with 10 mM CM-H2DCFDA (Life Technologies, Carlsbad, CA, USA, # C6827), which is a cell-permeable indicator for ROS. For the detection of mitochondrial ROS levels, the isolated IECs were incubated with Mitosox (M36008, Life Technologies) for 15 min following the manufacturer’s instructions. Subsequently, the fluorescence levels were quantified by flow cytometry. All flow data were analyzed by FlowJo software (FlowJo 10.7.1).

### 2.11. Colonic Explants

Whole colons were harvested from LRRK2 WT or LRRK2 G2019S KI mice, thoroughly rinsed with serum-free DMEM and weighed to determine their initial weight. The collected colon tissues were cut into 2 mm pieces and then cultured as explants in regular RPMI 1640 medium supplemented with 10% FBS, L-glutamine, penicillin and streptomycin, and placed in a standard cell culture incubator for 24 h. After the culture, the cell-free supernatants were obtained by centrifuging at 12,000× *g* for 10 min at 4 °C and stored in aliquots at −20 °C for further analysis.

### 2.12. Quantitative PCR

Whole colon tissues or cells were carefully preserved and homogenized using TRIzol reagent (Invitrogen, Waltham, MA, USA) to ensure optimal RNA extraction. The RNA extraction procedure was carried out following the manufacturer’s instructions, and the extracted RNA was promptly reverse transcribed into complementary DNA (cDNA). Quantitative PCR (Q-PCR) analysis was performed using SYBR Green Real-time PCR Master Mix on a Real-Time PCR System (Applied Biosystems, Waltham, MA, USA). The primer sequences utilized for the Q-PCR amplification are provided in Supplemental Table S2.

### 2.13. Immunohistochemistry

Formalin-fixed and paraffin-embedded colon sections or tumor samples were meticulously deparaffinized and rehydrated. Antigen retrieval was performed using citrate antigen retrieval buffer, followed by gradual cooling to room temperature. Following permeabilization and blocking steps, the sections were incubated overnight at 4 °C with primary antibodies, including anti-cyclin D1 (dilution, 1:200, CST), anti-p-STAT3 (dilution, 1:200, CST) and anti-Ki67 (dilution, 1:500, CST). Subsequently, the sections were incubated with fluorescence-conjugated secondary antibodies. The images were taken by a fluorescence microscope (Olympus, Tokyo, Japan, model DP74-CU).

### 2.14. Histological Analysis

For H&E staining, approximately 3 mm sections of colon tissues were meticulously fixed in 10% formalin or 4% paraformaldehyde and subsequently embedded in paraffin. The paraffin-embedded sections were then stained with H&E to facilitate histological analysis. Scoring of the sections was performed in a scale ranging from 0 to 10 by summarizing the scores of the severity of inflammation, the extent of inflammation and crypt damage as adapted from previous studies [51,52]. In brief, the severity of inflammation was scored on a scale of 0–3, with 0 indicating no inflammation, 1 denoting mild inflammation, 2 representing moderate inflammation and 3 indicating severe inflammation. The extent of inflammation was also scored on a scale of 0–3, with 0 indicating no inflammation, 1 denoting involvement of the mucosa, 2 representing involvement of both the mucosa and submucosa and 3 indicating transmural/muscularis/serosa involvement. Crypt damage was scored on a scale of 0–4, with 0 indicating no damage, 1 representing one-third crypt damage, 2 denoting two-thirds crypt damage, 3 indicating loss of crypts with surface and epithelium still present and 4 denoting complete loss of crypts and surface epithelium.

### 2.15. Subcellular Fractionation Protocol

Cytosol and mitochondria fragments were isolated using a Mitochondria/Cytosol Fractionation Kit (Abcam, Cambridge, UK, ab65320). In brief, cells were treated with 500 μL of fractionation buffer and incubated on ice for 15 min. To ensure cell lysis, the cell suspension was passed through a 27-gauge needle 10 times using a 1 mL syringe or until all cells were lysed. The lysed sample was then left on ice for an additional 20 min. The lysed sample was centrifuged at 3000 rpm for 5 min; the resulting pellet contained nuclei while the supernatant contained cytoplasm, membranes and mitochondria. Then, the supernatant was carefully transferred into a fresh tube and subjected to centrifugation at 8000 rpm for 5 min, so the resulting pellet contained mitochondria. The supernatant, which contained the cytoplasm and membrane fraction, was transferred into a fresh tube and stored for further analysis. The subcellular fractionation buffer used in the protocol included 20 mM HEPES (pH 7.4), 10 mM KCl, 2 mM MgCl_2_, 1 mM EDTA and 1 mM EGTA. Additionally, 1 mM DTT was added, and 1 piece of PI cocktail was added per 10 mL of buffer just before use.

### 2.16. Statistical Analysis

The *p*-values of weight loss comparison were determined by two-way ANOVA as specified in the figure legend. *p*-values for two group comparisons were determined by Student’s *t* test. Unless otherwise specified, all results are shown as mean ± SD. A *p*-value < 0.05 was considered significant.

## 3. Results

### 3.1. LRRK2 G2019S Promotes the Pathogenesis of Colitis-Associated Cancer

**Figure 1:**
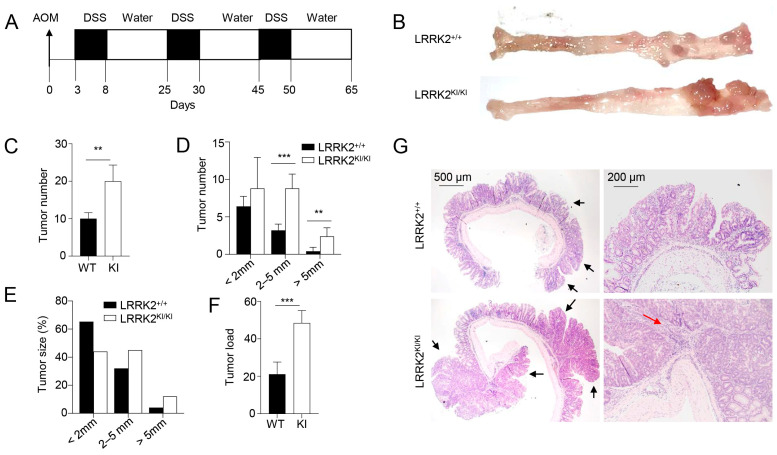
LRRK2 G2019S promotes the pathogenesis of colitis-associated colon cancer. (**A**) Schematic diagram for AOM/DSS-induced inflammatory carcinogenesis. We administered 8–12-week-old mice with AOM at a dose of 10 mg/kg mouse by intraperitoneal (IP) injection. Three days later, mice were given 2.5% DSS for 5 days, and then mice were rested for 16 days. This process was repeated for another two cycles starting on day 25 and day 45, respectively. Mice were euthanized on day 65 for analysis, as stated below. (**B**) Representative image of gross colons from LRRK2^+/+^ and LRRK2^KI/KI^ mice. (**C**) Tumor numbers in colons of LRRK2^+/+^ (WT) and LRRK2^KI/KI^ (KI) mice. (**D**) Tumor size distributions in colons of LRRK2^+/+^ and LRRK2^KI/KI^ mice. (**E**) Tumor size percentage in colons of LRRK2^+/+^ and LRRK2^KI/KI^ mice. (**F**) Average tumor load was determined by summing all tumor diameters for a given animal. (**G**) H&E staining of tumor sections from colons of LRRK2^+/+^ and LRRK2^KI/KI^ mice. Black arrows point to the tumors. The red arrow points to tumor cells invading the lamina propria. *p*-values were determined by Student’s *t* test, with *n* = 5 mice/group in each experiment. Data represent the mean ± SD. *** *p* < 0.01 and *** *p* < 0.001. Data are from one representative experiment out of three independent experiments.

Previous studies have provided evidence that individuals carrying the LRRK2 G2019S mutation are at an increased risk of developing cancers [21,22]. However, the direct impact of this mutation on cancer development has not been tested to date. Here, we utilized a mouse model of CAC [31,46] to test the role of LRRK2 G2019S in colon tumorigenesis in LRRK2 G2019S KI (LRRK2 KI) mice. In this model, mice receive a single treatment of AOM by intraperitoneal injection, followed by three cycles of DSS treatment in regular drinking water (Figure 1A). After AOM/DSS induction, both LRRK2 KI mice and WT controls developed colon tumors and the tumors were exclusively clustered in the distal colon (Figure 1B). Some of the LRRK2 KI mice exhibited severe rectal bleeding, diarrhea or weight loss toward the end of the treatment. Additionally, the average number of tumors per mouse in LRRK2 KI mice (20 ± 3.85) was about double that in WT counterparts (9.8 ± 2.56) (Figure 1C). The presence of LRRK2 G2019S also influenced the size of the tumors. Approximately 30.6% of the polyps developed in WT mice were small adenomas (2–5 mm diameter), in contrast with 45% in LRRK2 KI mice. Furthermore, 6.1% in WT mice versus 11% of polyps in LRRK2 KI mice developed into large tumors (>5 mm diameter) (Figure 1D,E). Similarly, the average tumor load, calculated by summing the diameters of all tumors in each mouse, was significantly higher in LRRK2 KI mice compared to WT mice (Figure 1F). Further histological analyses revealed that the majority of lesions formed in WT mice were either polypoid colonic tissue or tubular adenomas and most with low-grade dysplasia, featuring basally located, elongated, hyperchromatic nuclei. There was no lamina propria invasion. In contrast, the large tumors formed in LRRK2 KI mice exhibited extensive and confluent high-grade dysplasia, loss of nuclei polarity and lamina propria invasion, characteristic of adenocarcinoma (Figure 1G). Tumorigenesis can be divided into three mechanistic stages: initiation (genomic alteration), promotion (proliferation of genetically altered cells) and progression (tumor growth) [53]. Therefore, these data indicate that LRRK2 G2019S not only enhances tumor promotion but also increases tumor progression.

### 3.2. LRRK2 G2019S Promotes Inflammation and Cell Proliferation in Tumors

**Figure 2:**
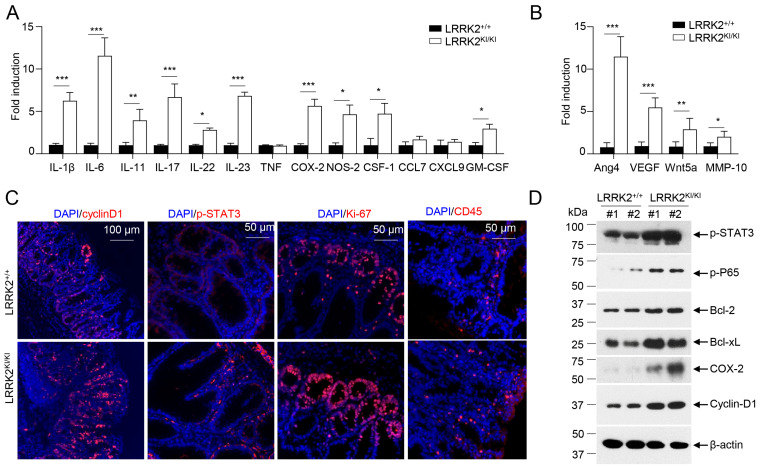
LRRK2 G2019S promotes inflammation and cell proliferation in colon tumors. At 65 days after AOM/DSS induction, LRRK2^+/+^ and LRRK2^KI/KI^ mice were euthanized, and colon tumor tissues were collected for the following analysis: (**A**) Real-time PCR analysis of inflammatory genes in colon tumors. (**B**) Real-time PCR analysis of pro-tumorigenic genes in colon tumors. (**C**) Fluorescence staining of tumor tissues with indicated antibodies and counterstained by DAPI. (**D**) Immunoblot analysis of key proteins involved in colon tumorigenesis as indicated. Lysates were prepared from colon tumor tissues of LRRK2^+/+^ and LRRK2^KI/KI^ mice. Numbers represent individual mouse from each group. *p*-values were determined by Student’s *t* test, with *n* = 5 mice/group. Data represent mean ± SD. * *p* < 0.05, ** *p* < 0.01 and *** *p* < 0.001. Data are from one representative experiment out of three independent experiments.

To elucidate the molecular mechanisms underlying the impact of LRRK2 G2019S on CAC development, we examined colon tumor tissues from AOM/DSS-treated LRRK2 KI and WT mice to assess the activation of various pro-tumor effectors. First, we compared the pro-inflammatory gene expression in the colon tumor tissues using real-time PCR. The result revealed much higher expression of inflammatory genes including IL-1β, IL-6, IL-11, IL-17, IL-23, COX-2, etc.; while expression of TNF, CCL7 and CXCL9 was comparable between the two groups (Figure 2A). Moreover, factors associated with tissue remodeling and angiogenesis, such as Ang4, VEGF, Wnt5a and MMP10, were also significantly upregulated in the LRRK2 KI mice (Figure 2B).

IL-1β has been shown to play an important role in CAC development via activating NF-κB [54]. IL-6, a well-established cytokine induced by IL-1β in intestinal epithelial cells (IECs) [55], has been shown to promote tumor progression in CAC through the activation of the oncogene STAT3 [56] in addition to IL-11 [57]. Consistent with these, we observed increased staining of phosphorylated STAT3 in the colon IECs of LRRK2 KI mice (Figure 2C). The activation of STAT3 or NF-κB upon AOM/DSS induction was expected to promote proliferation and survival of IECs, and then we tested the proliferation markers in the colon epithelium of LRRK2 KI and WT mice. Cyclin D1, which is a target gene of both STAT3 and NF-κB and critical for cell proliferation and survival, was highly expressed in the epithelium of LRRK2 KI mice compared to WT mice. The Ki-67 protein has been widely used as a proliferation marker for tumor cells. Consistently, we detected a higher number of Ki-67-positive cells in the colon epithelium of LRRK2 KI mice compared to WT controls (Figure 2C). In addition, we observed increased CD45+ leukocyte infiltration in the tumor tissue in the LRRK2 KI mice (Figure 2C). Consistent with gene expression analysis and immunohistochemical staining, immunoblot analysis revealed that p-STAT3, p-P65, Bcl-2, Bcl-xL and cyclin D1 levels in tumor tissues from KI mice were dramatically increased. Cox-2 is important to produce prostaglandin E2 (PGE2) during inflammation. Of note, PGE2, which is the most abundant prostanoid in colorectal cancer, promotes anti-tumor immune responses by inducing Treg and MDSCs [30] and promotes tumor initiation and growth by upregulating the expression of DNMT1 and DNMT3B [58]. The immunoblot assay suggested an increased level of COX-2 in the tumor tissues from LRRK2 KI mice (Figure 2D). Taken together, these results demonstrate that the activation of IL-1β-NF-κB and IL-6/IL-11-STAT3 pathways may potentially play pivotal roles in the promotion of tumorigenesis in the colons of LRRK2 KI mice.

### 3.3. LRRK2 G2019S KI Mice Are Highly Susceptible to DSS-Induced Colitis

**Figure 3:**
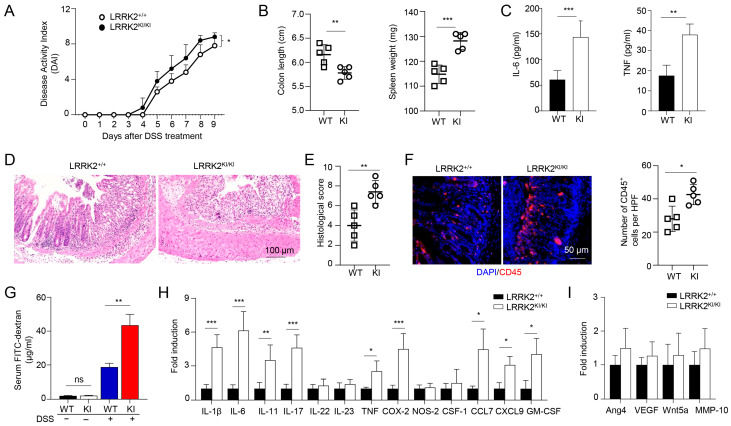
LRRK2 G2019S KI mice are highly susceptible to DSS-induced colitis. Acute colitis was induced in LRRK2^+/+^ (WT) and LRRK2^KI/KI^ (KI) mice with 2.5% DSS in drinking water for 7 days. Mice were euthanized on day 10. Colitis severity was shown by the following: (**A**) Disease activity index. (**B**) Colon length and spleen weight. (**C**) IL-6 and TNF levels in colon explant culture (100 mg colon tissue/mL medium) analyzed by ELISA. (**D**,**E**) H&E staining of colonic tissue and histology score. (**F**) Fluorescence staining of colon tissues with anti-CD45 antibody and counterstained by DAPI. (**G**) Gut permeability assay. Untreated or DSS-treated LRRK2^+/+^ and LRRK2 ^KI/KI^ mice were gavaged by FITC-dextran, 4 h later sera were collected, and FITC-dextran level was measured. ns: not significant. (**H**) Real-time PCR analysis of Inflammatory genes in the colon tissues. (**I**) Real-time PCR analysis of pro-tumorigenic genes in colon tissues. n = 5/group except panel A (*n* = 10/group). *p*-values were determined by two-way ANOVA in panel A and Student’s *t* test in other related panels. Data represent mean ± SD. * *p* < 0.05, ** *p* < 0.01 and *** *p* < 0.001. Data are from one representative experiment out of three independent experiments.

The association between chronic inflammation and the development of colorectal cancer has been extensively documented [59,60]. To unravel the mechanisms underlying the increased tumor promotion and progression observed in LRRK2 KI mice, we hypothesized that this mutation could enhance the susceptibility to DSS-induced colitis, thus promoting cancer pathogenesis. To test this, we utilized the DSS-induced colitis model to investigate the susceptibility of LRRK2 KI mice to intestinal inflammation. Both LRRK2 KI mice and WT control mice were subjected to 2.5% DSS treatment in drinking water for 7 consecutive days. Notably, LRRK2 KI mice exhibited an elevated disease activity index (Figure 3A). In addition, LRRK2 KI mice manifested more exacerbated colitis symptoms including a shortened colon length and increased spleen weight when compared with those in WT mice (Figure 3B). Analysis of supernatants from the colonic explant culture revealed much higher levels of the cytokines of IL-6 and TNF-α in LRRK2 KI mice compared with WT controls (Figure 3C). Additionally, histology analysis showed that LRRK2 KI mice displayed heightened gut damage and inflammatory cell infiltration following DSS treatment when compared with WT controls (Figure 3D,E). The enhanced inflammatory response in LRRK2 KI mice was associated with increased leukocyte infiltration in colon tissue (Figure 3F), and exacerbated impairment of intestinal epithelial integrity, as demonstrated by the increased leakage of FITC-dextran from the gastrointestinal tract into the systemic circulation (Figure 3G). Consistent with the observed tissue damage and inflammation, we also identified highly upregulated expression of pro-inflammatory cytokines and chemokines, such as IL-1β, IL-6, IL-11, IL-17, CXCL9, COX-2, etc., in the colons of LRRK2 KI mice compared with WT controls (Figure 3H). However, the expression of pro-tumorigenic genes including Ang4, VEGF, Wnt5a and MMP-10 was comparable in the LRRK2 KI and WT control groups (Figure 3I), suggesting that LRRK2 G2019S may promote tumor development primarily by promoting inflammation. Notably, we did not detect any difference in colon structure by H&E staining or pro-inflammatory cytokine expression by Q-PCR when the LRRK2 KI and WT controls had a homeostatic status without any treatment, suggesting that LRRK2 G2019S does not affect the baseline of gut inflammation. Taken together, our findings indicate that LRRK2 KI mice are more susceptible to DSS-induced colitis than WT counterparts, and LRRK2 G2019S may promote tumorigenesis through the promotion of inflammation.

### 3.4. Kinase Activity of LRRK2 G2019S Is Potentially Critical for Exacerbated Colitis and CAC

**Figure 4:**
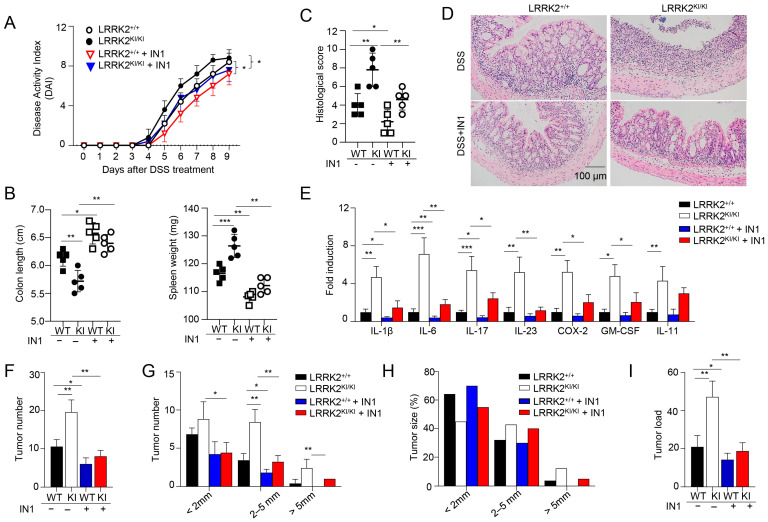
Kinase activity of LRRK2 G2019S is critical for exacerbated colitis and colitis-associated cancer. First, acute colitis was induced in LRRK2 G2019S^KI/KI^ (KI) and LRRK2^+/+^ (WT) control mice. LRRK2-IN-1(IN-1) inhibitors were used to treat both the wild-type (WT) and KI mice once a day at a dose of 100 mg/kg by i.p. injection, and mice treated with vehicles were included as controls as indicated. Mice were euthanized on day 9. Colitis severity was shown by the following: (**A**) Disease activity index. (**B**) Colon length and spleen weight. (**C**,**D**) H&E staining of colonic tissue and histological score. (**E**) Real-time PCR analysis of inflammatory gene expression as indicated in colon tissues. Second, colitis-associate cancer was induced in KI and WT controls. Mice were euthanized on day 65 for analysis, as stated below. (**F**) Tumor numbers in colons of indicated groups. (**G**) Tumor size distributions in colons of indicated groups. (**H**) Tumor size percentage in colons of indicated groups. (**I**) Average tumor load was determined by summing all tumor diameters for a given animal in indicated groups. *n* = 5 mice/group. Two-way ANOVA was used to determine *p*-values in panel A and Student’s *t*-test in other related panels. Data represent mean ± SD. * *p* < 0.05, ** *p* < 0.01 and *** *p* < 0.001. Data are from one representative experiment out of three independent experiments.

Previous studies have demonstrated the suppressive effects of the LRRK2 kinase inhibitor LRRK2-IN-1 on cytokine production in vitro and colitis in vivo [47]. To examine the potential role of LRRK2 kinase activity in DSS-induced colitis, we then investigated the impact of LRRK2-IN-1 treatment on colitis development in both LRRK2 KI mice and littermate controls using the DSS colitis model. Remarkably, the administration of LRRK2-IN-1 inhibited DSS-induced colitis in both the LRRK2 KI and WT groups (Figure 4). First, we found that the disease activity index was drastically reduced by the inhibitor not only in the LRRK2 KI groups but also in the WT groups (Figure 4A). Treatment with LRRK2-IN-1 alleviated the reduction in colon length (Figure 4B), which is indicative of the severity of colon inflammation. An enlarged spleen weight, indicating a system inflammation induced by DSS, was also relieved by this potent inhibitor (Figure 4B), suggesting that LRRK2-IN-1 played an important role in dampening the intestinal inflammation. Moreover, histology analysis revealed that LRRK2-IN-1 treatment alleviated colon damage (Figure 4C,D). Furthermore, the inhibition of LRRK2 kinase activity partially mitigated colonic inflammation at the molecular level, as detected by Q-PCR in both the LRRK2 KI and WT groups (Figure 4E). Consistent with these results, in the process of CAC induction by AOM/DSS, the inhibition of LRRK2 G2019S kinase activity by LRRK2-IN-1 also ameliorated the severity of colon cancer. As show in Figure 4F–I, the tumor number and tumor load in the colons of LRRK2 KI mice after LRRK2-IN-1 treatment became comparable with those in WT controls. In addition, the tumors with sizes of more than 2 mm were dramatically reduced after inhibitor treatment compared to those in LRRK2 KI mice with vehicle treatment. In total, these findings suggest that LRRK2 kinase activity plays a crucial role in the development of DSS-induced colitis, which indicates that targeting LRRK2 kinase activity provides a potential method for inhibiting the development of colitis and colitis-associated cancer.

### 3.5. LRRK2 G2019S Promotes Inflammasome Activation and Necrosis in the Gut Epithelium

**Figure 5:**
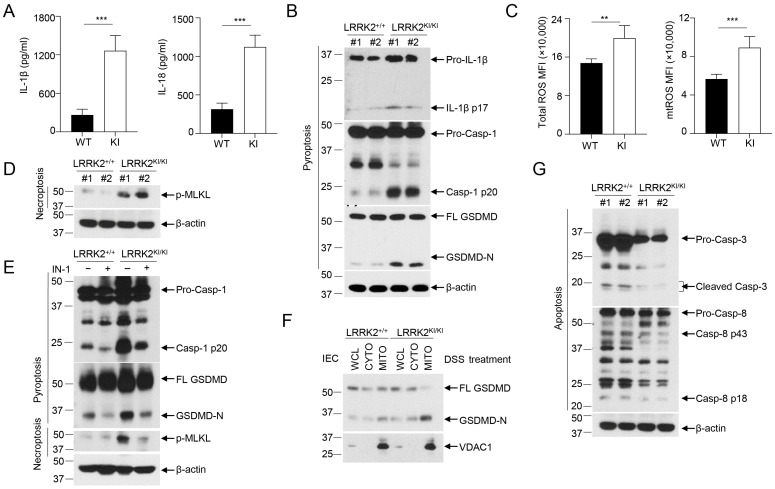
LRRK2 G2019S promotes inflammasome activation and necrosis in the gut epithelium. (**A**) IL-1β (left panel) and IL-18 (right panel) protein levels in the supernatants from a colon explant culture of LRRK2^+/+^ (WT) and LRRK2^KI/KI^ (KI) mice 9 days after DSS colitis induction were measured by ELISA as indicated. (B-G) IECs from LRRK2^+/+^ and LRRK2^KI/KI^ mice were isolated 9 days after DSS colitis induction and were collected for the following experiments: (**B**) Inflammasome activation in IECs was analyzed by immunoblot as indicated. (**C**) Mean fluorescence intensities (MFIs) of total ROS and mitochondrial ROS (mtROS) from IECs are shown. ROS levels were analyzed by flow cytometry. (**D**) Necroptosis in IECs were analyzed by immunoblot as indicated. (**E**) Inflammasome activation and necrosis in IECs was analyzed by immunoblot as indicated, where IN-1 means LRRK2-IN-1 inhibitor. (**F**) GSDMD-N levels in cellular fractions of IECs were analyzed by immunoblot as indicated. IECs were lysed, and then whole cell lysates (WCLs) were fractionated into cytoplasm (CYTO) and mitochondria (MITO). (**G**) Apoptosis in IECs was analyzed by immunoblot as indicated. *p*-values were determined by Student’s *t* test. *n* = 5 mice/group. Data represent mean ± SD. ** *p* < 0.01, *** *p* < 0.001. Data are from one representative experiment out of two independent experiments.

Inflammasome activation plays an important role in gut homeostasis and IBD [61]. We have recently demonstrated that LRRK2 is an upstream regulator of NLRC4 inflammasome activation [8]. Of note, gain-of-function mutations of NLRC4 result in hyperactivation of the inflammasome and lead to autoinflammation and enterocolitis [62,63]. These findings indicate that LRRK2 G2019S may play an important role in inflammasome activation in the DSS colitis model. Therefore, we determined the status of inflammasome activation in colon tissues from LRRK2 KI mice and WT controls after colitis induction. General markers for inflammasome activation include the production of mature/secreted IL-β and IL-18, caspase-1 cleavage and gasdermin D (GSDMD) cleavage, which leads to cell pyroptosis. First, we measured the secretion of IL-β and IL-18 in the supernatants of colonic explant cultures from LRRK2 KI and WT controls by ELISA, and we found that both IL-1β and IL-18 levels were much higher in supernatants from LRRK2 KI mice when compared with those from WT controls (Figure 5A). Second, we utilized immunoblotting to analyze the inflammasome activation in the gut epithelium by isolating IECs from the colons of LRRK2 KI mice and WT controls, and we observed increased cleavage of IL-1β, caspase-1 and GSDMD in the IECs from LRRK2 KI mice when compared with WT counterparts (Figure 5B), suggesting more robust inflammasome activation in the gut epithelium of LRRK2 KI mice after DSS treatment.

ROS have been found to be increased in IBD patients, and they are also a well-established factor in promoting inflammasome activation [64]. A recent study suggested that LRRK2 G2019S promotes mitochondrial ROS (mtROS) production in bone-marrow-derived macrophages (BMDMs) with transgenic LRRK2 G2019S expression [65]. Furthermore, increased mtROS mobilize cleaved GSDMD-N to the mitochondria and form pores in the membrane, which further enhance the release of mtROS. The excessive ROS in LRRK2 G2019S transgenic BMDMs promotes necroptosis instead of pyroptosis [65]. We then wondered whether LRRK2 G2019S promotes ROS production and necroptosis in the gut epithelium. Intriguingly, our data indicated an increase in both total ROS and mitochondrial ROS levels in IECs from LRRK2 KI mice when compared with WT controls after DSS treatment (Figure 5C). With that, we further tested the necroptosis of IECs from LRRK2 KI mice and WT controls by immunoblot analysis. We found a marked increase in phosphorylated MLKL (Figure 5D), suggesting elevated necroptosis in the gut epithelium of LRRK2 KI mice. Furthermore, we observed that LRRK2 inhibitor treatment ameliorated the inflammasome and necrosis activation in IECs after DSS treatment in both KI and WT mice (Figure 5E). Interestingly, we also observed the increased translocation of GSDMD to the mitochondria of the IECs in LRRK2 KI mice following DSS treatment (Figure 5F), suggesting that G2019S promotes GSDMD-N to locate in the mitochondria, and that this action may be universal and not limited to a specific cell type. In contrast to necroptosis and pyroptosis, we observed decreased apoptosis, characterized by cleaved caspase-3 and caspase-8 activation, in the gut epithelium of LRRK2 KI mice after DSS treatment (Figure 5G). Taken together, our data demonstrate that LRRK2 G2019S KI promotes inflammasome activation and necrosis in the context of DSS-induced colitis. These findings shed light on the underlying mechanisms through which LRRK2 G2019S may contribute to the pathogenesis of colon cancer.

### 3.6. GSDMD Inhibitors Ameliorated the Severity of Colitis in LRRK2 G2019S KI Mice

**Figure 6:**
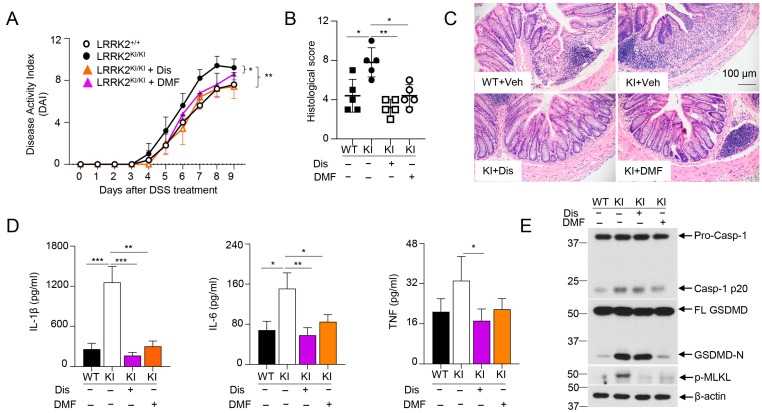
GSDMD inhibitors ameliorated the severity of DSS-induced colitis in LRRK2 KI mice. Acute colitis was induced in LRRK2^+/+^ (WT) and LRRK2^KI/KI^ (KI) mice with 2.5% DSS in drinking water for 7 days, and mice were euthanized on day 9. Two groups of LRRK2^KI/KI^ (KI) mice were treated with GSDMD inhibitors once a day during the DSS induction process. Disulfiram (Dis) was administered at a dose of 50 mg/kg by i.p. injection while dimethyl fumarate (DMF) was administered by oral gavage at 50 mg/kg. Colitis severity was shown by the following: (**A**) Disease activity index. (**B**,**C**) H&E staining of colonic tissue and histological score. (**D**) IL-1β, IL-6 and TNF levels in colon explant culture (100 mg colon tissue/mL medium) analyzed by ELISA. (**E**) Immunoblot analysis of proteins in the colon epithelial cells as indicated. – means not added, + means added. Two-way ANOVA was used to determine *p*-values in panel A and Student’s *t*-test in other related panels. *n* = 5/group. Data represent mean ± SD. * *p* < 0.05, ** *p* < 0.01 and *** *p* < 0.001. Data are from one representative experiment out of two independent experiments.

The data from Figure 5 as stated above indicated that LRRK2-mediated GSDMD activation might play crucial roles in DSS-induced colitis in LRRK2 KI mice. Intriguingly, we previously demonstrated that IECs are the major source of GSDMD in the gut mucosa of colitis mice. Furthermore, GSDMD expression is drastically upregulated in mucosal biopsies from IBD patients when compared with healthy controls. Of note, GSDMD deficiency attenuated the colitis severity induced by DSS compared to that with WT control mice [66]. Therefore, we hypothesized that GSDMD inhibitor treatment in LRRK2 G2019S KI mice will reduce DSS-induced colitis. Importantly, GSDMD inhibitors have shown great promise in treating autoinflammatory diseases [67]. Among these, two FDA-approved drugs, disulfiram and diroximel fumarate (DMF), which are used for the treatment of alcohol dependence and relapsing multiple sclerosis, respectively, have been proven to be efficacious GSDMD inhibitors [48,49]. To test this hypothesis, we induced colitis in both WT and LRRK2 KI mice. LRRK2 KI mice were treated with GSDMD inhibitors, either by disulfiram or DMF. Markedly, both inhibitors attenuated DSS-induced colitis in LRRK2 KI mice. First, the disease activity index was significantly reduced by two GSDMD inhibitors in KI mice (Figure 6A). Second, histology analysis revealed that GSDMD inhibitors alleviated colon damage and inflammation in KI mice (Figure 6B,C). GSDMD plays two essential functions, one is to execute cell pyroptosis and the other is to mediate IL-1β/IL-18 release. We then tested the two readouts in colon explants’ cultures after disulfiram or DMF treatment in KI mice along with IL-6 and TNF-α by ELISA. We found that the levels of IL-1β, TNF-α and IL-6 in the supernatants from inhibitor-treated groups were dramatically reduced compared those from vehicle-treated KI mice (Figure 6D). The levels of LDH and IL-18 were also decreased after inhibitor treatment (data not shown) in KI mice. While both GSDMD inhibitors are effective at GSDMD function suppression, the underlying mechanisms are different. Disulfiram inhibits GSDMD by preventing pore formation. However, DMF inhibits GSDMD through GSDMD succination, which prevents its interaction with caspases, thus limiting its processing and capacity to induce cell death [48,49]. To test the distinct impacts of disulfiram and DMF on GSDMD processing in vivo, we collected IECs from the colon after DSS colitis induction and analyzed the caspase-1 cleavage and GSDMD cleavage by Western blotting. Consistent with previous reports [48,49], immunoblot analysis demonstrated that disulfiram treatment did not affect the GSDMD cleavage. In contrast, DMF significantly prevented the GSDMD cleavage in IECs of KI mice (Figure 6E). Of note, both inhibitors attenuated the levels of p-MLKL, suggesting decreased necroptosis in GSDMD-inhibitor-treated LRRK2 KI mice (Figure 6E). Taken together, these results suggested that GSDMD plays critical roles in DSS-induced and LRRK2-mediated intestinal inflammation.

### 3.7. LRRK2 G2019S Promotes Inflammation and Cell Proliferation during the Early Stage of Tumorigenesis

**Figure 7:**
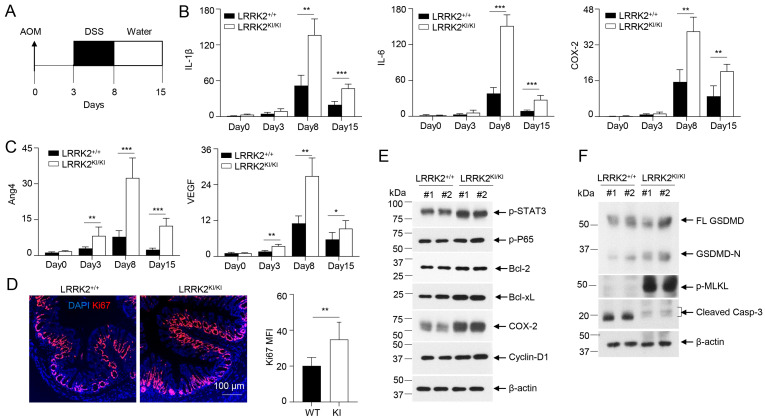
LRRK2 G2019S promotes inflammation and cell proliferation during early stage of tumorigenesis. (**A**) Schematic timeline for AOM and DSS treatment. (**B**) Real-time PCR of kinetic inflammatory gene expression in colon tissues from LRRK2^+/+^ and LRRK2^KI/KI^ mice as indicated. (**C**) Kinetic gene expression of Ang4 and VEGF in colon tissues from LRRK2^+/+^ and LRRK2^KI/KI^ mice was measured by real-time PCR. (**D**) Ki-67 staining of colon tissues from LRRK2^+/+^ (WT) and LRRK2^KI/KI^ (KI) mice 15 days after treatment. Mean florescence intensity (MFI) was quantified by Image J. (**E**) Immunoblot analysis of key proteins involved in colon tumorigenesis as indicated. Lysates were prepared from whole colon tissues of LRRK2^+/+^ and LRRK2^KI/KI^ mice 15 days after treatment. Numbers represent individual mouse from each group. (**F**) Immunoblot analysis of markers of cell death from colon epithelial cells of LRRK2^+/+^ and LRRK2^KI/KI^ mice 15 days after treatment. *n* = 3 mice/group. *p*-values were determined by Student’s *t*-test. Data represent mean ± SD. * *p* < 0.05, ** *p* < 0.01 and *** *p* < 0.001. Data are from one representative experiment out of two independent experiments.

To gain further insights into how LRRK2 G2019S contributes to increased tumor promotion and progression, we conducted a comparative analysis of the inflammation and proliferation status in the colon tissues of LRRK2 KI mice and WT control mice during the early stage of tumorigenesis following AOM and DSS treatment (Figure 7A). After AOM administration and the first cycle of DSS treatment, we found a much stronger induction of the inflammatory genes of IL-1β, IL-6 and COX-2 in colon tissues of LRRK2 KI mice compared to those in WT controls on day 8 and day 15 after AOM/DSS treatment, but these genes were not primed by AOM alone three days after its administration (Figure 7B). In contrast, the pro-tumorigenic genes Ang4 and VEGF were found to be more robustly expressed in the colons of LRRK2 KI mice three days after AOM treatment, and further increased in both groups after DSS treatment but more dramatically upregulated in LRRK2 KI mice (Figure 7C). Consistent with the enhanced expression of pro-inflammatory and pro-tumorigenic genes in the colon tissue of LRRK2 KI mice, immunofluorescent staining uncovered an increased number of Ki-67-positive cells in the colon epithelium of LRRK2 KI mice 15 days after AOM/DSS administration (Figure 7D), indicating elevated cell proliferation of IECs in the guts of LRRK2 KI mice during the early stages of AOM/DSS induction when compared with WT controls.

In the AOM/DSS model of CAC, AOM introduces mutation and genetic instability in the gut epithelium and DSS promotes inflammation in the tumor microenvironment, and together they induce robust colon tumorigenesis [31,46]. Consistent with this, LRRK2 G2019S mice did not develop colon tumors when subjected to a single AOM injection or repeated DSS treatment alone 65 days after treatment. Consistent with the hyper expression of IL-1β and IL-6 in the colon tissues of LRRK2 KI mice (Figure 7B), immunoblot analysis demonstrated increased p-STAT3 and p-P65 levels compared to those in WT controls (Figure 7E). Correspondingly, we observed elevated levels of COX-2 and cyclin D1 in the colon tissues of LRRK2 KI mice (Figure 7E). In the DSS-induced acute colitis model, we observed increased necrosis in the epithelium of LRRK2 KI mice when compared with WT controls (Figure 5B,D). We then considered whether LRRK2 G2019S promotes necrosis in the gut epithelium in the early stage of colon tumorigenesis. Immunoblot analysis revealed higher levels of GSDMD-N and p-MLKL in the IECs from LRRK2 KI mice early after AOM/DSS treatment (Figure 7F), suggesting increased necrosis. In contrast, we observed decreased cleaved caspase-3 in the IECs from LRRK2 KI mice, which may be explained by the results showing that hyperactivation of NF-κB and STAT3 in LRRK2 G2019S IECs upregulated the levels of Bcl-2 and Bcl-xL (Figure 7E), which play critical roles in the anti-apoptosis response. Therefore, our data suggested that the overall impact of LRRK2 G2019S is to promote IEC proliferation in the early stage of colon tumorigenesis despite the fact that it can also promote necrosis. Necrosis promoted by LRRK2 G2019S may play a critical role in enhancing pro-tumorigenic inflammation.

## 4. Discussion

Numerous studies have indicated that patients with Parkinson’s disease (PD) who carry the LRRK2 G2019S mutation face elevated risks of developing cancers, including colorectal cancer [21,22]. Despite these findings, the underlying mechanisms remain largely elusive. Our study contributes to the latest understanding by demonstrating that LRRK2 G2019S promotes colon tumorigenesis in a mouse model of colitis-associated cancer (CAC). Through the genetic study of LRRK2 G2019S KI mice, we further elucidated that LRRK2 G2019S promotes intestinal inflammation in a DSS-induced colitis model. Given the established positive correlation between colitis and colorectal cancer, our data suggest that LRRK2 G2019S-mediated colitis potentially exacerbates the pathogenesis of colorectal cancer in PD patients carrying this mutation. However, we are aware of the limitations of this study. One limitation of this study is that LRRK2 G2019S KI mice do not spontaneously develop Parkinsonism; therefore, it is imperative to develop a PD model with the LRRK2 G2019S mutation and further evaluate its susceptibility to tumorigenesis in the future. Another limitation is that we used the LRRK2-IN-1 kinase inhibitor to suppress LRRK2 kinase activity in vivo. While this inhibitor has been proven to be potent and selective [68], off-target effects have also been reported [69]. Therefore, it is critical to further validate our results in the future with more potent LRRK2 kinase inhibitors or with LRRK2 kinase-dead KI mice.

Although LRRK2 has been identified as the gene most associated with PD, meta-GWAS has also revealed LRRK2 to be a major susceptibility gene for Crohn’s disease (CD) [11,12,13]. Of note, recent exome-sequencing analyses further revealed novel LRRK2 variants shared by both PD and CD, including LRRK2 N2081D [40]. Notably, LRRK2 G2019S, the most common genetic determinant of PD, is located in the kinase domain, as is the N2081D variant. These observations prompted us to speculate that LRRK2 G2019S might promote intestinal inflammation and colitis, which is critical for the pathogenesis of colon cancer. Using the DSS-induced colitis model, several groups have tested the role of LRRK2 in intestinal inflammation. Liu et al. found that LRRK2 ablation promotes colitis in the DSS-induced colitis model [70]. In contrast with this, a later study by another group suggested that high expression of LRRK2 by using LRRK2 transgenic mice also promotes intestinal inflammation when using the same animal model, and they found that the LRRK2 kinase inhibitor attenuates colitis severity in both LRRK2 transgenic mice and WT controls [47]. Consistent with the latter study, Lin et al. used LRRK2 G2019S transgenic mice and found that LRRK2 G2019S promotes intestinal inflammation in a DSS-induced chronic colitis model by upregulating TLRs, NF-κB and pro-inflammatory cytokines, especially TNF-α [71].

One limitation of using transgenic mice is that the site of integration of the transgene into the genome can significantly affect tissue specificity and levels of transgene expression [72]. To address this limitation, we utilized LRRK2 G2019S KI mice and revealed that LRRK2 G2019S KI mice displayed notable weight loss, shortened colon length, increased spleen weight and heightened gut damage compared to WT littermates, suggesting that LRRK2 G2019S promotes colitis in the DSS-induced acute colitis model. Furthermore, the inhibition of the kinase activity of LRRK2 mitigated colitis severity in both KI mice and WT controls, underscoring the crucial role of LRRK2 kinase activity in intestinal inflammation. These novel findings were published in bioRxiv in June 2023 [73]. Subsequently, we demonstrated that the LRRK2 G2019S-GSDMD signaling axis is pivotal for the increased colitis in LRRK2 KI mice, which is now integrated into this report alongside the findings previously reported in bioRxiv [73]. Later, the Baekelandt V group reported that LRRK2 G2019S KI mice exhibited more severe colitis compared to WT controls [74], consistent with our findings in bioRxiv [73]. It is noteworthy that while the approaches used to induce colitis between the Baekelandt V group and our group differ, our results using G2019S KI mice, along with the two previous studies using LRRK2 [47] or LRRK2 G2019S transgenic mice [71], support the notion that gain-of-kinase activities of LRRK2 promote intestinal inflammation.

Although several studies have described LRRK2 kinase activity as playing a critical role in DSS-induced acute and chronic colitis, the underlying mechanisms remain elusive. Inflammasomes, crucial regulators of gut homeostasis and intestinal disorders such as inflammatory bowel disease (IBD) [61], have garnered attention in this context. Despite research on inflammasome activation and its downstream effectors in experimental colitis models like the DSS-induced colitis model yielding inconsistent or even opposing results at times, clinical studies suggest a positive correlation between inflammasome hyperactivation and colitis, particularly IBD [61,75].

Gene polymorphisms of NLRP3 and IL-18 have been implicated in conferring susceptibility to IBD [76,77,78]. Mononuclear cells isolated from the lamina propria of active colonic lesions in IBD patients produce elevated levels of IL-1β and IL-18 [79,80,81], with colon IL-1β levels correlating with disease activity [82,83]. Single-cell immune profiling has revealed IL-1β signatures in macrophages/monocytes from inflamed intestinal tissues of IBD patients [84]. Additionally, mutations in the IL-18R1–IL-18RAP locus are associated with susceptibility to IBD [11,85,86]. However, the most direct evidence supporting the positive correlation between inflammasome hyperactivation and IBD stems from the identification of autoinflammation with infantile enterocolitis (AIFEC). AIFEC arises from inborn errors of NLRC4, leading to hyperactivation of the NLRC4 inflammasome and driving disease pathogenesis [62,63]. We previously discovered that LRRK2 is critical for the activation of the NLRC4 inflammasome, and its kinase activity is important for this function and host defense [8]. In the current DSS-induced colitis model, we found that LRRK2 G2019S promotes inflammasome activation, resulting in elevated production of IL-1β and IL-18 in the gut epithelium. However, whether this occurs via the NLRC4 inflammasome or other pathways remains uncertain and warrants further investigation in future studies.

Upon the activation of the inflammasome, cells undergo pyroptosis, alongside the production of mature IL-1β and IL-18. Pyroptosis, akin to necroptosis, is a form of programmed necrosis [87]. Consistent with the hyperactivation of the inflammasome in the gut epithelium of DSS-treated LRRK2 G2019S KI mice, we observed enhanced GSDMD cleavage, suggesting robust pyroptotic cell death in the intestinal epithelium. Furthermore, we also observed increased necroptosis. However, we did not see increased apoptosis, suggesting that LRRK2 G2019S does not promote PANoptosis [88]. Interestingly, a recent study proposed that LRRK2 G2019S disrupts mitochondrial homeostasis and alters cell death pathways in macrophages by facilitating the translocation of cleaved GSDMD to the mitochondrial membrane. Consequently, the G2019S variant favors necroptosis over pyroptosis [65]. Notably, we observed increased GSDMD-N localization in the mitochondrial fraction of IECs from LRRK2 G2019S mice after DSS treatment, implying that LRRK2 G2019S promotes MLKL-mediated necroptosis not only in macrophages but also in epithelial cells. This suggests that enhanced necroptosis in the LRRK2 G2019S gut epithelium may result partly from GSDMD-N translocation to the mitochondria promoted by the G2019S mutation. Consistent with the phenotype in LRRK2 G2019S macrophages, elevated oxidative stress was observed in LRRK2 G2019S IECs, indicated by increased ROS production. ROS can trigger inflammasome activation, potentially perpetuating inflammation in the intestine of LRRK2 G2019S KI mice. IEC death is a hallmark of IBD, with elevated apoptosis and necroptosis of IECs correlating positively with inflammation severity in IBD [89,90,91]. IEC death can lead to gut barrier dysfunction, microbial dysbiosis and systemic pathogen dissemination [92,93,94]. Furthermore, necrotic cell death releases DAMPs such as ATP, HMGB1, IL-1α, hyaluronan and IL-33, which exacerbate intestinal inflammation [95]. Although our results indicate that LRRK2 G2019S promotes intestinal inflammation at multiple levels, our data underscore the pivotal role of GSDMD in LRRK2 G2019S-mediated intestinal inflammation in LRRK2 KI mice. Treating G2019S KI mice with the GSDMD inhibitors disulfiram and DMF significantly attenuated DSS-induced colitis, comparable to WT control mice. It is imperative to assess whether MLKL inhibitors can mitigate intestinal inflammation in these models in future studies, given our findings that LRRK2 G2019S promotes necroptosis. These data further support our previous report that GSDMD contributes to the colitis pathogenesis in mice following DSS treatment [66]. In summary, the current study suggests that LRRK2 G2019S promotes colon cancer through LRRK2–GSDMD signaling axis-mediated intestinal inflammation.

It is well-established that patients with inflammatory bowel disease (IBD) face an increased risk of developing colorectal cancer due to sustained intestinal inflammation [24,25,28,29]. Inflammation fosters colon carcinogenesis through various pathways and at multiple levels [30]. Oxidative stress induced by inflammation can initiate tumorigenesis by causing DNA damage, while increased oxidative stress can, in turn, exacerbate inflammation [96]. Furthermore, inflammation can compromise the integrity of the gut barrier and disrupt the microbiota, influencing colon tumorigenesis [97]. Several inflammation-triggered signaling pathways, notably NF-κB and STAT3, are known to play pivotal roles in colon tumorigenesis [98,99,100,101]. Our data suggest that the LRRK2 G2019S mutation enhances the production of IL-1β, IL-6 and IL-11 in the colon tumor microenvironment, thereby activating the NF-κB and STAT3 signaling pathways. Consequently, we observed elevated phosphorylation levels of STAT3 and p65 in LRRK2 G2019S colon tumors, accompanied by upregulation of downstream molecules such as Bcl-xL, cyclin D1 and COX-2. However, the precise mechanisms by which LRRK2 G2019S mediates these distinct signaling pathways and contributes to colon tumorigenesis warrant further exploration.

In conclusion, our study sheds light on the molecular mechanisms linking the LRRK2 G2019S mutation to colon carcinogenesis. We propose that LRRK2 G2019S promotes intestinal inflammation, creating an inflammatory microenvironment conducive to the development of colorectal cancer observed in patients with LRRK2 G2019S-associated Parkinson’s disease. Targeting LRRK2 kinase activity may emerge as a novel therapeutic strategy for colon cancer patients, particularly those harboring the G2019S mutation. As mentioned early, Takagawa T, et al. [47] found that LRRK2 gene overexpression promotes DSS-induced colitis; therefore, as an important future direction, it is imperative to explore whether this overexpression can also promote colitis-associated cancer, and if colon cancer patients have increased expression of LRRK2.

## Data Availability

All data are available upon request.

## Supplementary Materials

The following supporting information can be downloaded at: https://www.mdpi.com/article/10.3390/cells13070565/s1, Table S1: Antibody list (all the antibodies were diluted at a 1:1000 ratio before use). Table S2: Primer list.

## Abbreviations

AOM: azoxymethane; DSS: dextran sodium sulfate; GSDMD-N: gasdermin D N-terminal fragment; MLKL: mixed-lineage kinase domain-like protein; STAT3: signal transducer and activator of transcription 3; CAC: colitis-associated cancer; PD: Parkinson’s disease; IBD: inflammatory bowel disease; CD: Crohn’s disease; LRRK2: leucine-rich repeat kinase 2; LRRK2 KI: LRRK2 knock in; LRRK2 WT: LRRK2 wild type; DAI: disease activity index; H&E: hematoxylin and eosin; IECs: intestinal epithelial cells; PGE2: prostaglandin E2; MDSCs: myeloid-derived suppressor cells; COX-2: cyclooxygenase-2; Q-PCR: quantitative-polymerase chain reaction; ROS: reactive oxygen species; BMDMs: bone-marrow-derived macrophages; DMF: dimethylformamide; LDH: lactate dehydrogenase.
