# Supplementary material for: LRRK2 G2019S Promotes Colon Cancer Potentially via LRRK2–GSDMD Axis-Mediated Gut Inflammation
Source: Cells. 2024 Mar 23;13(7):565. doi: 10.3390/cells13070565 (PMC11011703; doi:10.3390/cells13070565)
Supplement: Supplementary file 1 [file cells-13-00565-s001.zip › cells-2904046-supplementary.pdf]

Supplemental Table S1. Antibody list (all the antibodies were diluted at a 1:1000 ratio before use**)**

**Antibodies**	**Catalog number**	**Company**
p-STAT3	9145T	Cell Signaling Technology (CST)
P-P65	3033T	CST
P-Bcl-2	2827S	CST
Bcl-XL	2764S	CST
COX-2	12282S	CST
Cyclin-D1	55506T cyclinD1	CST
β-actin	3700S	CST
IL-β	AF-401-NA	R&D
Caspase1	AG-20B-0042-C100	AdipoGen
GSDMD	ab209845	Abcam
Caspase3	9661	CST
Caspase8	8592S	CST
p-MLKL	91689S	CST
VDAC1	55259-1-AP	Proteintech
Ki-67	12202	CST

Supplemental Table S2. Primer list

Primer name	Primer sequence
IL-1β F	ACTGTTTCTAATGCCTTCCC
IL-1β R	TGGTTTCTTGTGACCCTGA
IL-6 F	TAGTCCTTCCTACCCCAATTTCC
IL-6 R	TTGGTCCTTAGCCACTCCTTC
IL-11 F	CTGACGGAGATCACAGTCTGGA
IL-11 R	GGACATCAAGTCTACTCGAAGCC
IL-17 F	CCTCACACGAGGCACAAGTG
IL-17 R	CTCTCCCTGGACTCATGTTTGC
IL-22 F	GCTTGAGGTGTCCAACTTCCAG
IL-22 R	ACTCCTCGGAACAGTTTCTCCC
IL-23 F	CCTTCTCCGTTCCAAGATCCT
IL-23 R	ACTAAGGGCTCAGTCAGAGTTGCT
TNF F	GACCCCTTTACTCTGACCCC
TNF R	AGGCTCCAGTGAATTCGGAA
COX2 F	GCGACATACTCAAGCAGGAGCA
COX2 R	AGTGGTAACCGCTCAGGTGTTG
NOS-2 F	GAGACAGGGAAGTCTGAAGCAC
NOS-2 R	CCAGCAGTAGTTGCTCCTCTTC
CSF-1 F	GCCTCCTGTTCTACAAGTGGAAG
CSF-1 R	ACTGGCAGTTCCACCTGTCTGT
CCL7 F	CAGAAGGATCACCAGTAGTCGG
CCL7 R	ATAGCCTCCTCGACCCACTTCT
CXCL9 F	CAGAACCTCCCACGTAGCTTTC
CXCL9 R	GCTCTGAAGATGGGATCAAGTTAATA
GM-CSF F	GGCCTTGGAAGCATGTAGAGG
GM-CSF R	GGAGAACTCGTTAGAGACGACTT
Ang4 F	GGTTGTGATTCCTCCAACTCTG
Ang4 R	CTGAAGTTTTCTCCATAAGGGCT
VEGF F	CTGCTGTAACGATGAAGCCCTG
VEGF R	GCTGTAGGAAGCTCATCTCTCC
Wnt5a F	GGAACGAATCCACGCTAAGGGT
Wnt5a R	AGCACGTCTTGAGGCTACAGGA
MMP10 F	TGCTGCCTATGAGGCTCACAAC
MMP10 R	GGAGGAAAACCGAGAGTGTGGA
